# Development of insecticide-impregnated polyester/cotton blend fabric and assessment of their repellent characteristics against *Cimex lectularius* and dengue vectors *Aedes albopictus* and *Aedes aegypti*

**DOI:** 10.1186/s13071-023-05740-1

**Published:** 2023-04-09

**Authors:** Ajay Kakati, Amartya Banerjee, Parikshit Das, Buddhadeb Saha, Danswrang Goyary, Sanjeev Karmakar, Sumit Kishor, Yangchen D. Bhutia, Pronobesh Chattopadhyay

**Affiliations:** Division of Pharmaceutical Technology, Defence Research Laboratory (DRL), DRDO, Tezpur, 784001 Assam India

**Keywords:** Vector-borne diseases, Dengue, Insecticide-impregnated fabric, Hematophagous insects

## Abstract

**Background:**

Personal protection measures using insecticide-treated fabric is one of the most effective strategies to prevent the bites of hematophagous insects. Many countries have had success treating fabrics with pyrethroids on an individual level.

**Methods:**

In the current study, a new combination of insecticides, alpha-cypermethrin (ACP) and deltamethrin (DET), has been impregnated on fabric composed of a 50:50 blend of polyester and cotton. Residual and morphological analysis was performed along with the evaluation of physical parameters. Biological evaluations were performed to check the repellency, knockdown, and mortality of insecticide-impregnated fabric (IIF) against bed bugs (*Cimex lectularius)* using Petri plate assay and mosquitoes (*Aedes aegypti* and *Aedes albopictus)* using cone bioassay.

**Results:**

The results showed the repellency of IIF to be 56.6% for *C. lectularius* and a knockdown percentage of 53.3% and 63.3% for *Ae. aegypti* and *Ae. albopictus*, respectively. A > 80% mortality was found for both species of mosquitoes up to 20 cycles of washing with no significant difference (*P* > 0.05). From high-performance liquid chromatography (HPLC) analysis, the reduction in the contents of ACP and DET after subsequent washes can be correlated with the overall decrease in bioefficacy. ACP and DET remaining in unit gram of fabric after 20 wash cycles were found to be 5.4 mg and 3.1 mg, respectively. By examining the fabric’s surface morphology using scanning electron microscope (SEM) and utilizing energy-dispersive x-ray (EDX) analysis, it was possible to identify the presence of insecticides that were adhered to the fabric. Differential scanning calorimetry (DSC) showed distinctive endothermic peak of insecticide at 98.3 ºC, whereas no change in thermal behavior was observed from thermo-gravimetric analysis (TGA). Furthermore, the physical attributes of IIF provide conclusive evidence for its firmness.

**Conclusion:**

All experimental findings were consistent with the potential use of IIF as a bed bug- and mosquito-repellent fabric to be used against hematophagous infestations. This fabric can serve as a potential strategy to control vector-borne diseases like dengue, malaria, trench fever, etc.

**Graphical Abstract:**

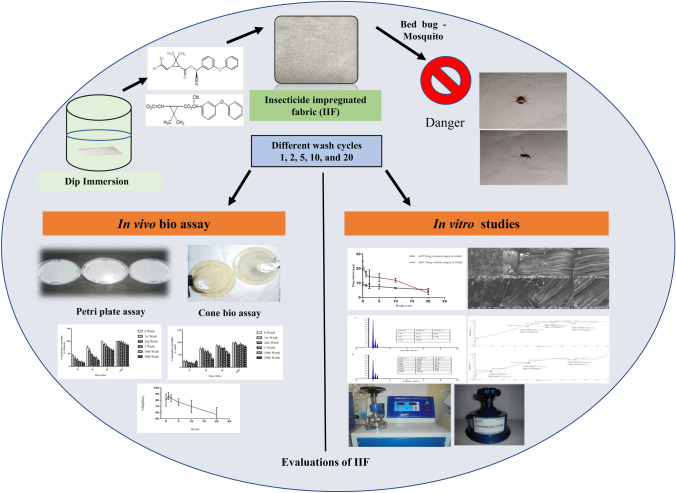

**Supplementary Information:**

The online version contains supplementary material available at 10.1186/s13071-023-05740-1.

## Background

Dengue, malaria, Japanese encephalitis, and filariasis are mosquito-borne illnesses that threaten more than 2 billion people worldwide, predominantly in tropical countries [1, 2]. Recent investigations indicate that *Aedes* spp. mosquitoes have possible significance in disease transmission in India [3–5]. *Aedes aegypti* and *Ae. albopictus* are the two most common vectors known to transmit several viral diseases like dengue, chikungunya, yellow fever and Zika [6–9]. In India, 63,280 cases and 44 fatalities caused by dengue were documented in 2022. Since 31 August 2022, 32,653 cases and 32 fatalities have occurred. The media reported a rapid rise in dengue cases in some other states such as Delhi, Uttar Pradesh, Assam, and West Bengal [10]. The most established way to manage insect vector-borne illnesses is the use of repellents [11, 12]. Many commercial insect repellents are unpleasant and ineffectual [13]. A good insect repellent must be long-lasting and effective against all species. The ideal repellent would prevent bites from a wide variety of insect species, be effective for a long time period, cause no skin or mucous membrane irritation, have no systemic toxicity, and be greaseless and odorless [14].

Pyrethroids are synthetically modified plant-based insecticides that differ from the natural analog, pyrethrum, which is obtained from *chrysanthemum* flowers [15]. Synthetic pyrethroids were first developed in the 1970s, introducing a new era of selective, highly effective, and environmentally friendly insecticides [1]. When deltamethrin was first released onto the market, it was reported to be 100 times more potent than DDT and had the advantage of not building up in the environment [3, 5]. Among the most commercially used pyrethroids, alpha-cypermethrin (ACP) and deltamethrin (DET) are already in use for the management of various insects [2]. Pyrethroids are safe and less toxic to mammals because of their rapid metabolism and large surface area; however, it is 2250 times more toxic to insects because of their smaller size, low body temperature, and excess expression of sensitive sodium channels [16, 17]. If we discuss its mechanism, among the most proposed theories for its repellency, locomotor excitation upon skin contact is considered to be most profound [3, 12]. The insecticidal effect also occurs via spatial contact mainly causing paralysis of the organism by preventing the voltage-gated Na^+^ channel from repolarizing in the axonal membrane [11].

There have been numerous attempts using various methods to impregnate pyrethroids on fabric. In general, the three most common methods of impregnation are the absorption method, which involves individually treating fabrics by dipping or spraying, the incorporation method, also referred to as “Eulanisierung,” which involves binding insecticides to wool or silk fibers using heat and salt gradients, and the polymer coating method, which is accomplished by specifically polymerizing pyrethroids onto fabrics prior to the tailoring process [18].

Hematophagous insects are those that feed on mammalian blood, such as bed bugs and mosquitoes. These ectoparasites have a long history of having a profound detrimental effect on human civilization. They are known to spread viruses, bacteria, and protozoans, three essential disease-causing organisms [19, 20]. All racial and socio-economic categories are affected by their infestations. The rate of bed bug infestation on human environments has dramatically increased over the past 2 decades as a result of growing international transportation as well as their resistance to chemical insecticides, raising worries about their detrimental effects [21].

Thus, this study has been designed to improve the personal protection by treating a polyester cotton blend fabric with a combination of pyrethroids, i.e., ACP and DET, that is effective against both mosquitoes and bed bugs. Combining cotton and polyester makes the fabric less prone to piling and static. The main advantage of polyester-cotton (PC) blends is that they are cheap and durable; being wrinkle-free, they also do not require ironing [22]. Insecticides like DDT are not suitable for treating fabrics because they act too slowly and allow insects to make contact and escape before they pick up a lethal dose [23–25]. The literature suggests that the suspension concentrate of ACP and DET is already used as acceptable treatment for insecticide-treated nets [26]. The bio-efficacy of developed fabric was confirmed by performing in vivo Petri plate assay and cone bioassay. The residual content after consequent wash cycles, surface morphological studies, and physical parameters were also considered to support the bioassay and quality attributes of the developed fabric for prevention of vectors, especially *Ae. aegypti* and *Ae. albopictus* species of mosquitoes and *Cimex lectularius* categories of common bed bugs that can transmit disease pathogens.

## Materials and methods

### Materials

Insecticides (alpha-cypermethrin and deltamethrin) were procured from Sigma Aldrich, St. Louis, MO, USA; polyvinyl acetate (PVA) (HiMedia Laboratories Pvt. Ltd., Mumbai, India); acetone (Fisher Scientific, Mumbai, India); acetonitrile, methanol, and water (Merck Life Science Pvt. Ltd., Mumbai, India) were procured and used in this study.

### Experimental methodology

#### Preparation of insecticide-impregnated fabric (IIF)

The dipping method was used for the impregnation of insecticides into the fabric. The fabric is a blend of polyester and cotton (50:50). Briefly, 1% w/w of ACP and 2% w/w of DET were dissolved in a pre-mixed solution of acetone with 4% w/v PVA (as a binder). Then, the fabric was dipped into the above solution for 30 min and air dried [18]. The prepared fabric was used for further analysis.

#### Washing procedure of the fabric samples

The washing technique recommended by the World Health Organization (WHO) was followed with a slight modification [27, 28]. Briefly, 400 ml of 2 g/l detergent solution in distilled water with pH adjusted to 10–11 (0.1 N HCl) was prepared. Samples were soaked and kept in a water bath at 30 ºC for 10 min at 155 rpm. After that, the fabric samples were rinsed twice in deionized water and dried at room temperature. The same procedure was repeated for further washes [29].

#### Residual analysis of IIF using high-performance liquid chromatography (HPLC)

##### Insecticide extraction procedure from the fabric sample

In this experiment, the ultrasonication method was used for the extraction of insecticides from fabric samples. Briefly, fabric samples were cut into ~ 1 g (8 cm × 8 cm) randomly from three different areas. It was chopped and soaked in a conical flask containing 7 ml extraction solvent (acetonitrile). Then flasks were heated at 75 °C in a water bath for 20 min with continuous shaking. Then, it was allowed to cool down to room temperature. Furthermore, the conical flasks were placed in an ultrasonic water bath and sonicated for 20 min at 30 °C. The supernatant was collected, filtrated using nylon filter (0.22 µm), and injected into HPLC for further analysis [30, 31].

##### Preparation of calibration curve and insecticidal residual analysis

Individual stock solutions of 1000 µg/ml were prepared for ACP and DET in acetonitrile. The calibration curves were prepared by diluting stock solutions with final concentrations of 2, 4, 6, 8, and 10 µg/ml. The chromatographic separations were performed on Thermo Scientific HPLC (Dionex Ultimate 3000, GmbH, Germany) coupled with Agilent reverse phase column (C_18_), 3.5 µm, and 4.6 × 250 mm with variable wavelength detector. For determination of maximum wavelength (λmax), 10 µg/ml solutions of each standard insecticide were scanned from 200–400 nm in a UV–VIS spectrophotometer (Cecil Instrumentation Services Ltd. C≡7200 series). The chromatographic experiments were performed under isocratic elution according to Additional file 1: Table S1. The amounts of ACP and DET contained in the extraction solvents were quantified according to their respective calibration curve generated using HPLC.

#### Surface morphology study using SEM and EDX

The surface morphologies of the IIF and control fabric were examined using a scanning electron microscope (SEM), JEOL JSM-6390LV, at an accelerating voltage of 15 kV. Representative electron micrographs were obtained, and the individual particles in a selected area were counted with data magnification of 50×, 200×, 500×, and 1000×. Scanning electron microscope (SEM) equipped with energy-dispersive x-ray (EDX) spectroscopy for surface elemental composition analysis was utilized for this purpose [32].

#### DSC-TGA analysis

Differential scanning calorimetry (DSC) and thermo-gravimetric analysis (TGA) were conducted using a PerkinElmer STA 6000 apparatus using samples of 2.5 mg of control fabric and 2.6 mg of IIF placed in a micro-perforated ceramic pan for internal pressure control to completely remove the water and other volatile substances from the testing pans. The test was conducted starting from an initial temperature of 30 °C to 600 °C at 10 °C/ min rise under 10 ml/min N_2_ flux [33].

#### Physical properties of fabrics

##### Flexural rigidity

The fabric bending length is calculated using a stiffness tester (Sri Balaji Chemicals & Instruments, Ashok Vihar, Delhi, India). At first, the overhanging length was obtained when the tip of the specimen (25 × 200 mm) had just reached a plane passing though the edge of the platform and was inclined at an angle of 41.5° below the horizontal. Then, flexural rigidity was calculated from the value of the bending length and weight per meter square area of the fabric (GSM) [34].

1 $${\text{Flexural rigidity }}\left( {\mu {\text{Nm}}} \right) \, = \, 9.807 \times 10\hbox{-}^{ - 6} \times {\text{W}} \times {\text{C}}^{3}$$

where W = fabric weight in grams per meter square area and C = bending length of specimen in mm.

##### Bursting strength (BS)

Bursting strength of the fabric was measured using a diaphragm hydraulic bursting strength tester (Sri Balaji Chemicals & Instruments, Ashok Vihar, Delhi, India). Three circular fabric specimens (≥ rubber diaphragm area 100 cm^2^) were taken from three different places on the whole fabric sample and tested by applying the force via diaphragm with an increasing order until the specimens burst. The results were reported in the form of mean ± standard deviation. A similar procedure was followed for the control fabric as well as for the IIF after several washes.

##### Bursting index (BI)

The bursting index is calculated as bursting strength per GSM of the fabric samples.

2 $${\text{BI}} = {\text{ BS }}\left( {{\text{KN}}} \right)/{\text{GSM }}\left( {{\text{g}}/{\text{m}}^{{2}} } \right)$$

##### Tex

Ten yarn samples were taken from both warp and weft directions (each 50 cm), and individual weights were recorded. The Tex values were calculated with the help of the following formula.

3 $${\text{Tex}} = {\text{Individual sample weight}} \left( {\text{g}} \right) \times \frac{100000}{{50}}$$

##### pH

Three pieces of fabric samples (0.5 cm × 0.5 cm) along with 100 ml distilled water were taken for fabric pH testing. All samples were placed in distilled water and shaken for 1 h. pH was measured using a digital pH meter (Oakton EUTECH Instruments), and values were recorded [35]. Studies were performed in triplicate, and results were reported as mean ± standard deviation.

#### In vivo studies

##### Petri plate assay

Plastic Petri plates of 8.5 cm diameter and 1.4 cm height were used to quickly evaluate the comparative repellency of untreated and IIF against bed bugs (*C. lectularius*) [maintained at Defence Research Laboratory (DRL), DRDO, Assam; maintained at room temperature of 25–28 °C, humidity 60 ± 15%, and 12:12 h photo period)]. Petri plate surface was divided into two equal halves; one half was covered with IIF and the other with control fabric. Ten bed bugs (7 days unfed adults) were released in the center of each Petri plate. The numbers of bed bugs present on each side of the plates were recorded after 24 h and calculated for percentage repellency [36]. Experiments were performed after the 0, 1st, 2nd, 5th, 10th, and 20th wash cycles in triplicate, and results were reported as mean ± standard deviation. The percentage repellency was calculated using the following formula.

4 $${\text{\% Repellency}} = \left( {\frac{{{\text{N}}_{{\text{C}}} - {\text{N}}_{{{\text{IIF}}}} }}{{{\text{N}}_{{\text{T}}} }}} \right) \times 100$$

Here, N_C_ = number of bed bugs on control fabric, N_IIF_ = number of bed bugs on IIF, and N_T_ = total number of bed bugs.

##### Cone bioassay

Mosquitoes (*Ae. aegypti* and *Ae. albopictus*) were reared at the Defence Research Laboratory, DRDO, Assam; maintained in a temperature range of 25–28 ºC, relative humidity 60 ± 15%, and 12:12 h photo-period. Larval stages were maintained in plastic trays (5 l) with (1:1) yeast powder and PUREPET™, Abis Exports (I) Pvt. Ltd., Chhattisgarh, India, as larval food source. Adult mosquitoes emerged from pupae were then maintained in wooden cages (30 × 30 × 30 inches) and provided with cotton soaked in 10% sugar solution.

The cone bioassay was performed according to WHO 2005 protocol. Ten non-blood-fed 2–5-day-old females were exposed to IIF and control fabric under a standard WHO cone for 3 min [37, 38]. Later, the exposed mosquitoes were transferred to a small cup (6.5 × 8.5 cm) with access to sugar solution and observed for their knockdown at 15, 30, and 60 min followed by mortality after 24 h of exposure [39]. Experiments were performed after the 0, 1st, 2nd, 5th, 10th, and 20th wash cycles in triplicate, and results were reported in the form of mean ± standard deviation.

## Results

The results showed that alpha-cypermethrin and deltamethrin-impregnated PC blend fabric was effective in repelling bedbugs (*C. lectularius*) and mosquitoes (*Ae. aegypti* and *Ae. albopictus*), also maintaining its various physical properties like stiffness, GSM, bursting strength, bursting index, etc., at different time intervals after different cycles of washings.

### Insecticide residual analysis of IIF by HPLC after different wash cycles

**Fig. 1:**
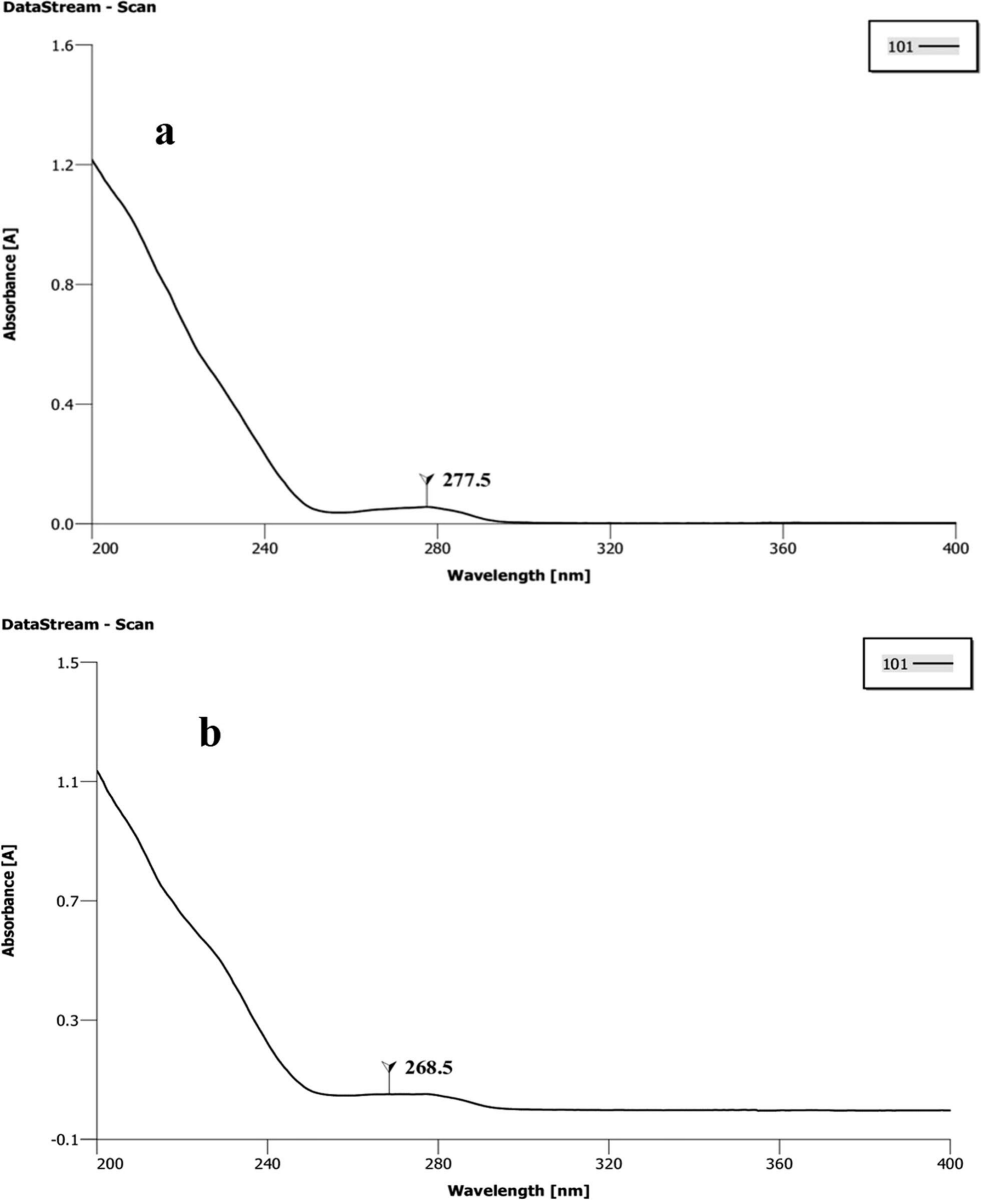
Determination of λmax for alpha-cypermethrin (ACP) (**a**) and deltamethrin (DET) (**b**)

**Fig. 2:**
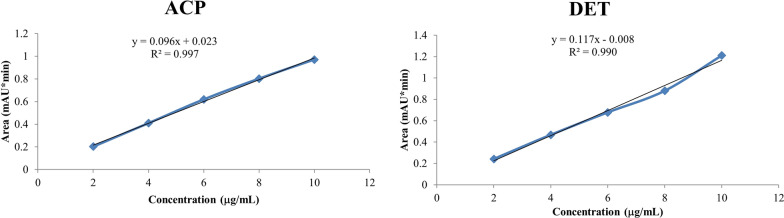
Standard curve of alpha-cypermethrin (ACP) (**a**) and deltamethrin (DET) (**b**)

**Fig. 3:**
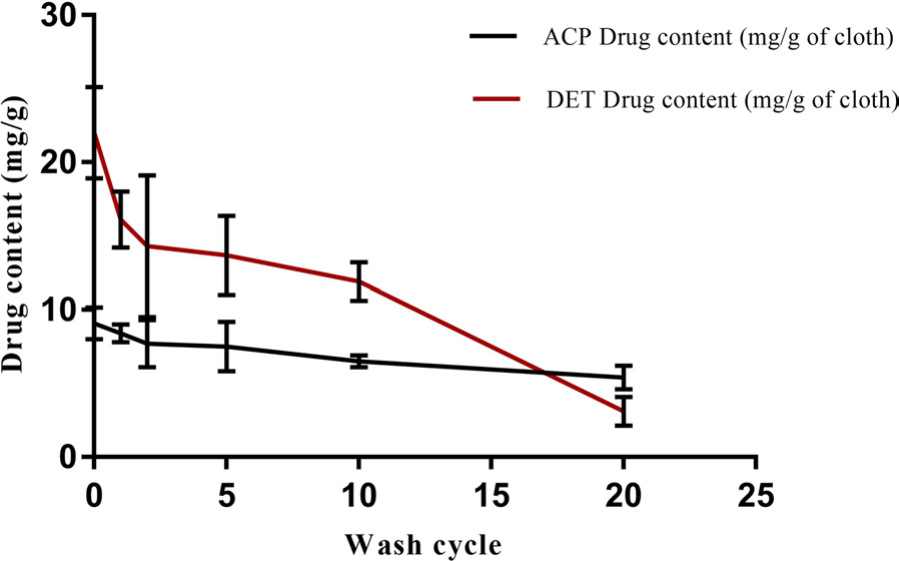
Insecticidal residual analysis of alpha-cypermethrin (ACP) and deltamethrin (DET) at different wash cycles: 0, 1, 2, 5, 10, and 20. Each value is represented in terms of mean ± standard deviation (*n* = 6)

The results of wavelength maxima for ACP and DET were found to be 277 nm and 268 nm, respectively (Fig. 1). Various aliquot concentrations were used to make the standard curves (Fig. 2). The retention times were 5.8 min and 5.0 min for ACP and DET, respectively. From the standard curves obtained, the calculations for insecticide residues were done, and the results for 0, 1st, 2nd, 5th, 10th, and 20th washes were reported in Table 1 and graphically representated in Fig. 3.

**Table 1:** Residual analysis of deltamethrin (DET) and alpha-cypermethrin (ACP) at different wash cycles

Wash cycle	Insecticide content in mg/g of fabric (*n* = 6)	
	DET	ACP
Zero wash	22.0 ± 3.1	9.1 ± 1.1
1st wash	16.1 ± 1.9	8.4 ± 0.6
2nd wash	14.3 ± 4.8	7.7 ± 1.6
5th wash	13.7 ± 2.7	7.5 ± 1.7
10th wash	11.9 ± 1.3	6.5 ± 0.4
20th wash	03.1 ± 1.0	5.4 ± 0.8

Results are expressed in terms of mean ± standard deviation (*n* = 6)

### SEM and EDX analysis of control fabric and IIF

**Fig. 4:**
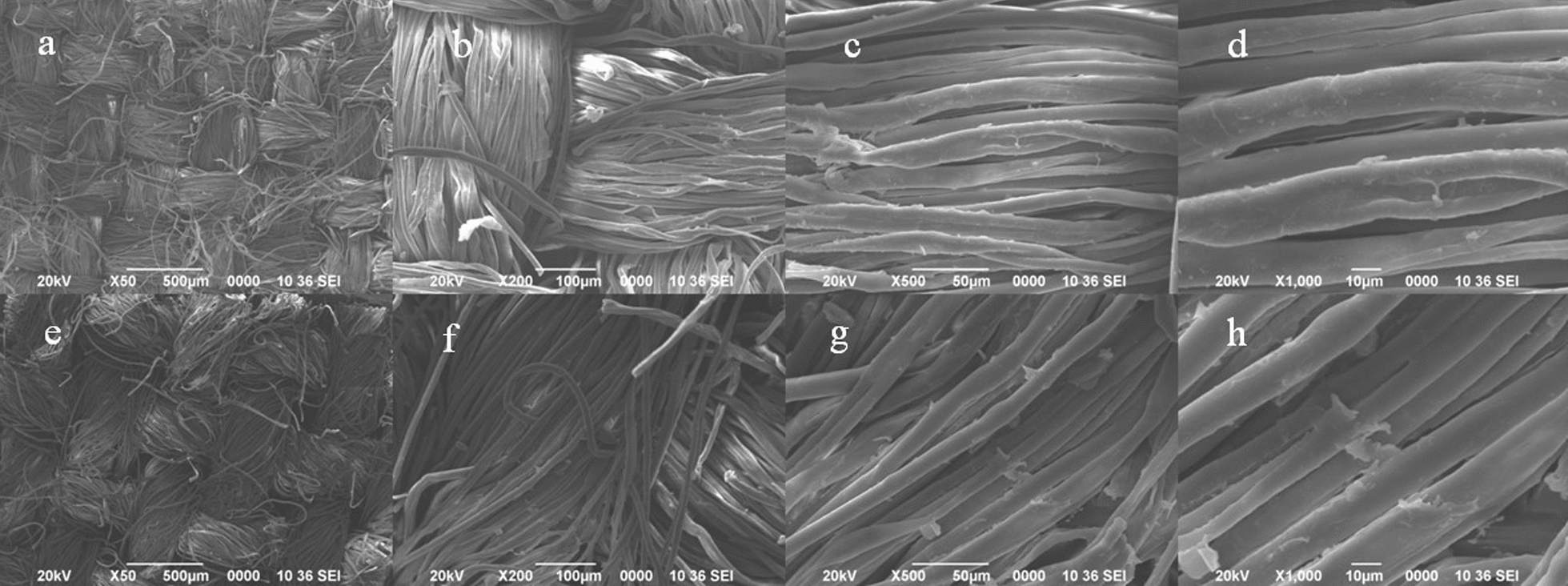
Scanning electron microscopy (SEM) images of control fabric (**a**–**d**) and insecticide-impregnated fabric (IIF) (**e**–**h**) at a magnification of 50×, 200×, 500×, and 1000×, respectively

**Fig. 5:**
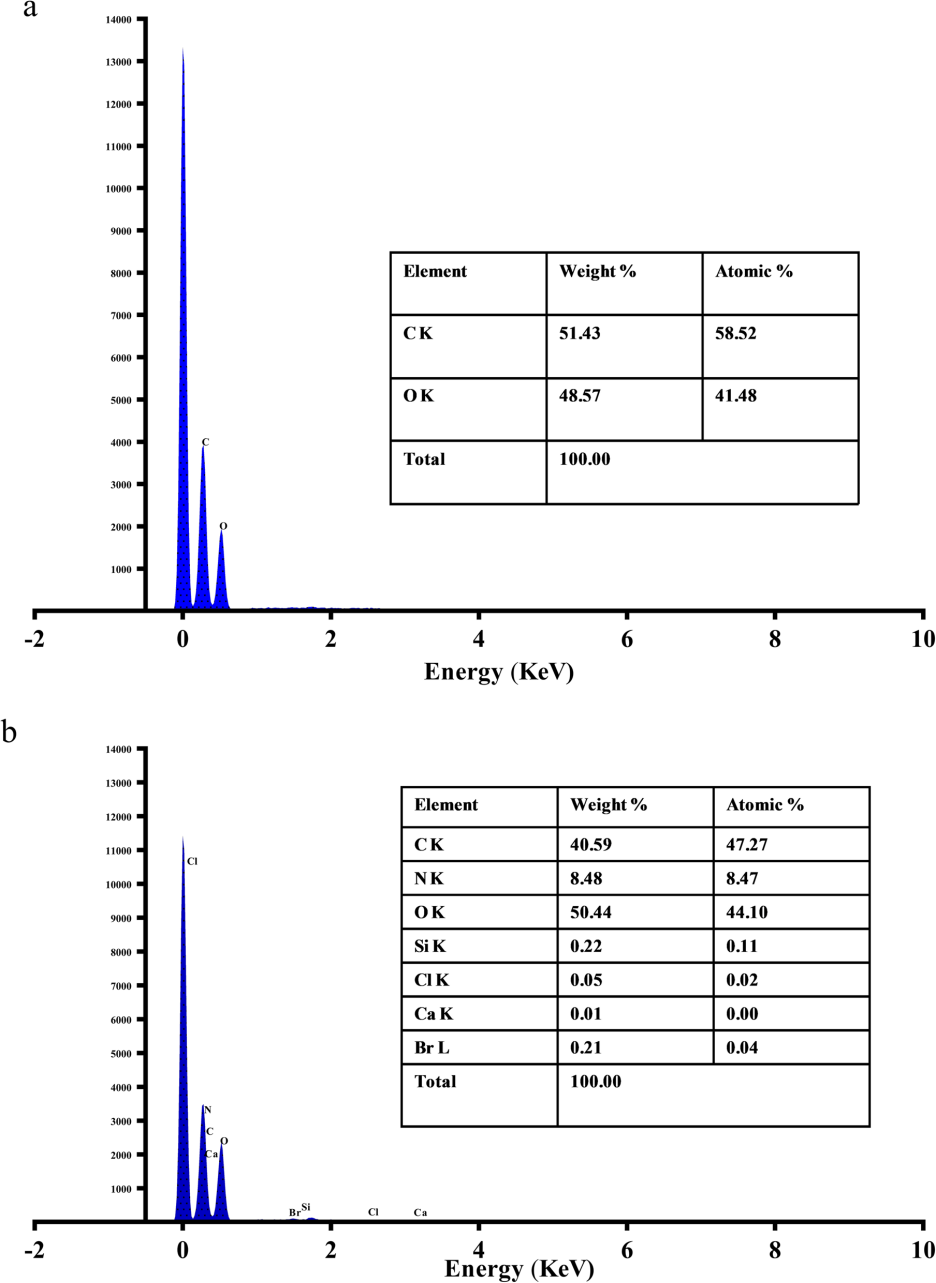
Energy-dispersive x-ray (EDX) spectra of control fabric (**a**) and insecticide-impregnated fabric (IIF) (**b**)

SEM was used to study the structural morphology of the fabric samples. The results have been shown for the control fabric as well as IIF. The samples were scanned with separate magnification at 50×, 200×, 500×, and 1000×. The SEM micrographs of control fabric and IIF are shown in Fig. 4. A clear distinction can be made on IIF that shows depostion of particles on the surface of the fabric, which are indicated by arrowheads. Additionally, the EDX spectra have been shown for both the control and IIF (Fig. 5). The EDX spectrum of control fabric shows the presence of only carbon and oxygen, whereas IIF shows the presence of carbon, oxygen, nitrogen, chlorine, and bromine.

### DSC-TGA analysis of control fabric and IIF

**Fig. 6:**
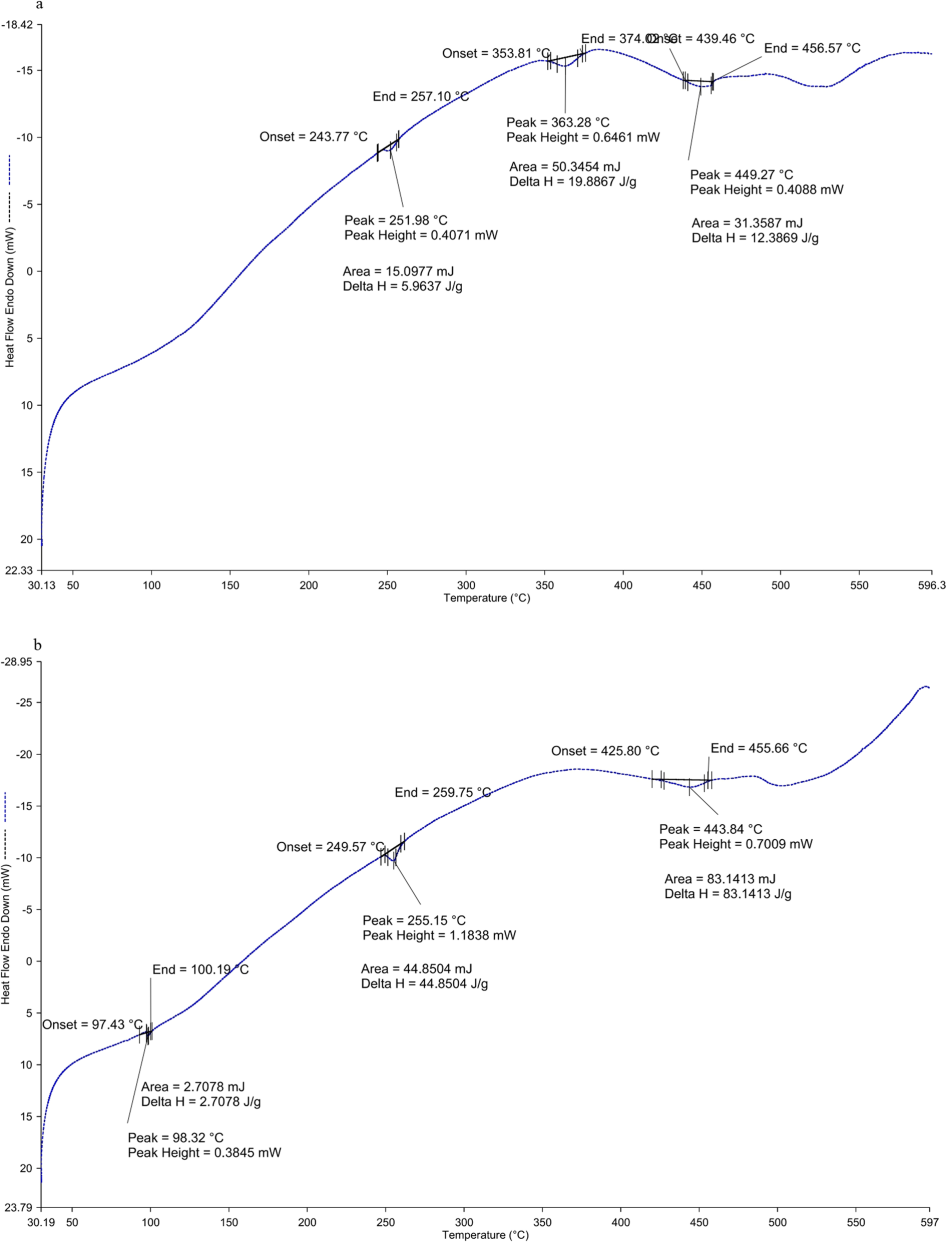
Differential scanning calorimetry (DSC) curve of control fabric (**a**) and insecticide-impregnated fabric (IIF) (**b**)

**Fig. 7:**
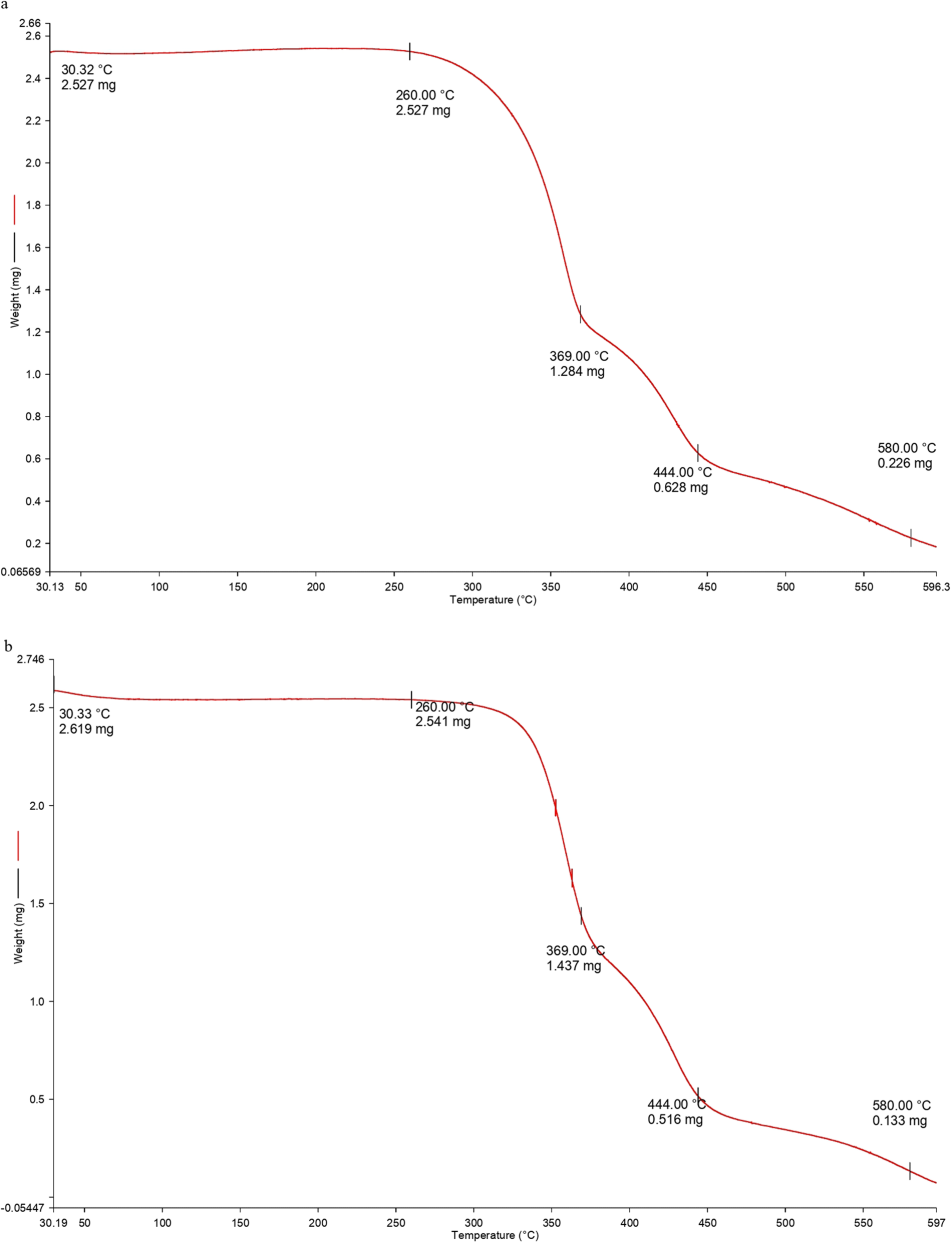
Thermogravimetric analysis (TGA) curve of control fabric (**a**) and insecticide-impregnated fabric (IIF) (**b**)

DSC-TGA analysis of control fabric and IIF has been shown in Figs. 6 and 7. The DSC thermogram of control fabric and IIF showed characteristic endothermic peaks. For control fabric, it showed peaks at 252 °C, 363 °C, and 450 °C, and for IIF, the endothermic peaks were found at 98.3 °C, 255.5 °C, and 443.8 °C. The TGA curve also showed a similar curve pattern for both control fabric and IIF. For control fabric, initial mass loss was observed after 260 °C. The mass loss up to 369 °C was 49.2%. A second mass loss was observed after 369 °C to 444 °C, which was 75.1%. As the temperatures rose to 600 ºC, final mass loss was found to be 91.0%. For IIF, initial mass loss was observed after 260 ºC. The mass loss to 369 °C was 43.4%, and a second mass loss was observed after 369 °C to 444 °C, which was 80.7%. As the temperatures rose to 600 °C, the final mass loss was 94.8%.

### Physical properties of control fabric and IIF

The results of physical parameters are presented in the form of mean ± standard deviation in Table 2. The fabric GSM and flexural rigidity were found to increase after insecticide impregnation. For control fabric, the values were 169.09 ± 0.85 g/m^2^ and 21.81 ± 2.52 µNm, whereas the same for IIF at zero wash were 172.50 ± 1.41 g/m^2^ and 49.80 ± 2.92 µNm, respectively; this further reduced to 169.36 ± 0.65 g/m^2^ and 12.96 ± 1.62 µNm for IIF after the 20th wash. The pH of the fabric was found to be near neutral within 7.03 ± 0.12 to 7.47 ± 0.06. The bursting strength and bursting index for control fabric were 15.07 ± 0.47 KN and 0.0891 ± 0.0032 KN.m^2^/gm whereas the same for IIF at zero wash were found to be 15.10 ± 0.6KN and 0.0876 ± 0.004 KN.m^2^/gm, respectively. This further reduced to 13.76 ± 0.72 KN and 0.0813 ± 0. 0044 KN.m^2^/gm at the 20th wash.

**Table 2:** Physical properties of fabric for control and insecticide-impregnated fabric (IIF) at different wash cycles

No. of washes	Fabric GSM (g/m^2^)	Flexural rigidity (µNm)	Bursting strength (KN)	Bursting index (KN.m^2^/gm)	Fabric pH
Control	169.1 ± 0.9	21.8 ± 2.5	15.1 ± 0.5	0.0891 ± 0.0032	7.07 ± 0.06
Wash 0	172.5 ± 1.4	49.8 ± 2.9	15.1 ± 0.6	0.0876 ± 0.0042	7.47 ± 0.06
Wash 1	172.4 ± 1.7	28.9 ± 3.3	14.7 ± 0.5	0.0855 ± 0.0027	7.43 ± 0.06
Wash 2	172.3 ± 1.1	23.6 ± 1.0	14.7 ± 0.4	0.0851 ± 0.0018	7.43 ± 0.06
Wash 5	172.2 ± 0.9	21.6 ± 2.3	14.7 ± 0.2	0.0856 ± 0.0015	7.40 ± 0.10
Wash 10	171.4 ± 1.1	14.1 ± 0.6	14.6 ± 0.8	0.0852 ± 0.0042	7.33 ± 0.12
Wash 20	169.4 ± 0.7	12.7 ± 1.6	13.8 ± 0.7	0.0813 ± 0.0044	7.03 ± 0.12

Each value is represented in terms of mean ± standard deviation (*n* = 3)

^*^Actual yarn count (Tex): 28.75, thickness (mm): 0.22, fabric type: plain woven fabric

### Petri plate assay for repellency of bed bugs

**Fig. 8:**
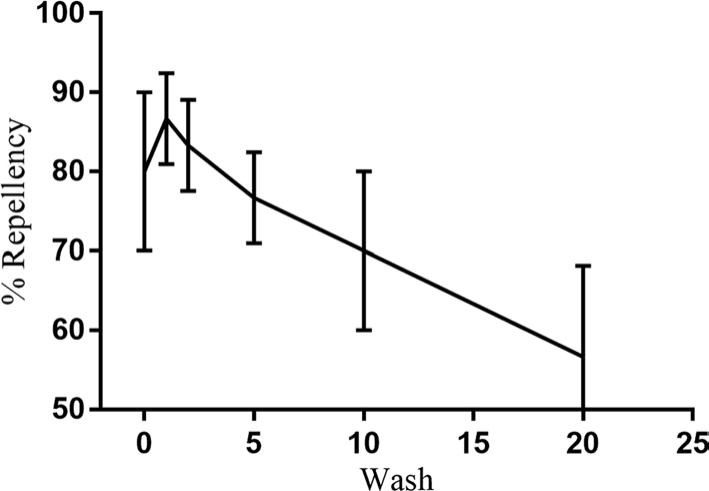
Petri plate assay of insecticide-impregnated fabric (IIF) against *Cimex lectularius*. Values are represented in terms of mean ± standard deviation (*n* = 3)

The results of Petri plate assay are represented in Table 3. At zero wash, the percentage repellency of IIF against bed bugs (*C. lectularius*) was 80.0% after 24 h of exposure, which gradually decreased to 56.7% after the 20th wash. The results are graphically represented in Fig. 8.

**Table 3:** Petri plate repellency assay of *Cimex lectularius*

No. of washes	Percentage repellency after 24 h
0	80.0 ± 10.0
1	86.7 ± 5.8
2	83.3 ± 5.8
5	76.7 ± 5.8
10	70.0 ± 10.0
20	56.7 ± 11.5

The results are expressed as mean ± standard deviation (*n* = 3)

### Cone bioassay for knockdown and mortalities of mosquitoes

**Fig. 9:**
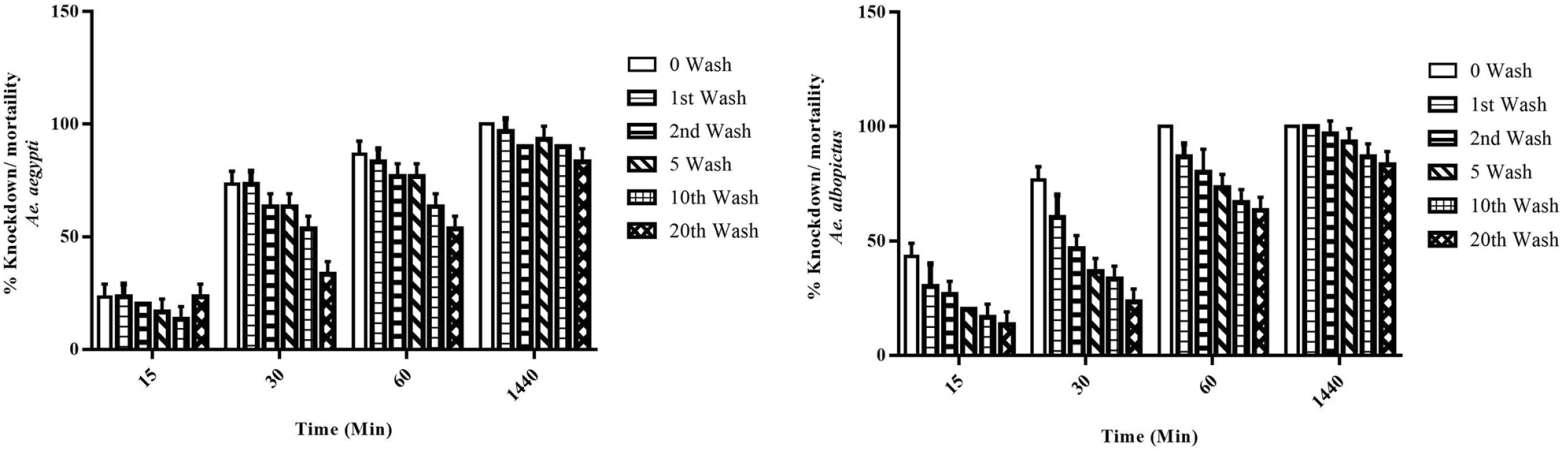
Cone bioassay of insecticide-impregnated fabric (IIF) at different wash cycles against *Aedes aegypti* mosquitoes (**a**) and *Ae. albopictus* (**b**). The percentage knockdown/mortality observed up to 24 h (1440 min). Each value is represented as mean ± standard deviation. The values were non-significant, *P* > 0.05 (one-way ANOVA followed by Tukey’s multiple comparison test)

The results of cone bioassay for the percent knockdown of *Ae. aegypti* and *Ae. albopictus* mosquitoes exposed to IIF after different cycles of washings at different time points are shown in Fig. 9. IIF without any washing could produce a percentage knockdown of about 23.3%, 73.3%, and 86.6% for *Ae. aegypti* mosquitoes and 43.3%, 76.6%, and 100% for *Ae. albopictus* mosquitoes at 15, 30, and 60 min post-exposure, respectively. Also, a percentage knockdown of > 50% and > 60% post-exposure (60 min) was observed in case of *Ae. aegypti* and *Ae. albopictus* mosquitoes respectively up to 20 cycles of washings. A mortality rate of around 80.0% was observed for both after 24 h of exposure. The mortality pattern of *Ae. aegypti* and *Ae. albopictus* mosquitoes exposed to IIF has been shown in Table 4. The results were statistically evaluated using one-way ANOVA followed by Tukey’s multiple comparison test using GraphPad Prism 7.00.

**Table 4:** Percentage knockdown and mortality at different time intervals using cone bioassay. Results are represented as mean ± standard deviation (*n* = 3)

No. of washes	*Aedes aegypti*				*Aedes albopictus*			
	Time				Time			
	Knockdown%			Mortality%	Knockdown%			Mortality%
	15 min	30 min	60 min	1440 min	15 min	30 min	60 min	1440 min
0	23.3 ± 0.6	73.3 ± 0.6	86.6 ± 0.6	100.0 ± 0.0	43.3 ± 5.8	76.7 ± 5.8	100.0 ± 0.0	100.0 ± 0.0
1	23.3 ± 0.6	73.3 ± 0.6	83.3 ± 0.6	96.7 ± 0.6	30.0 ± 10.0	60.0 ± 10.0	86.7 ± 5.8	100.0 ± 0.0
2	20.0 ± 0.0	63.3 ± 0.6	76.6 ± 0.6	90.0 ± 1.0	26.7 ± 5.8	46.7 ± 5.8	80.0 ± 10.0	96.7 ± 5.8
5	16.7 ± 0.6	63.3 ± 0.6	76.6 ± 1.2	90.0 ± 0.0	20.0 ± 0.0	36.6 ± 5.8	73.3 ± 5.8	93.3 ± 5.8
10	13.3 ± 0.6	53.3 ± 0.6	63.3 ± 0.6	90.0 ± 0.0	16.7 ± 5.8	33.3 ± 5.8	66.7 ± 5.8	86.7 ± 5.8
20	3.3 ± 0.6	36.6 ± 0.6	53.3 ± 0.6	80.0 ± 0.0	13.3 ± 5.8	23.3 ± 5.8	63.3 ± 5.8	83.3 ± 5.8
Statistical analysis	*P*-value = 0.908 *F-value *= 0.298				Statistical analysis	*P*-value = 0.670* F*-value = 0.643		

## Discussion

Many insect repellent formulations have been documented and marketed to date, but only a few are available that can show promising effective repellency throughout the complete shelf life. Unlike traditional formulations, which demand topical administrations and require extra efforts, this study was focused upon a unique fabric-oriented impregnation method as an alternate method to resist those disease spreading vectors in a more effortless and convenient way. We demonstrated that IIF is effective enough to repel lab-reared *Ae. albopictus* and *Ae. aegypti mosquitos* and also has sufficient repellency against laboratory-reared common bed bugs (*C. lectularius*) up to 20 launderings. According to recent research studies, bed bugs can also be effective vectors for infections including *Bartonella quintana* and *Trypanosoma cruzi* [40]. *Aedes* spp. are widely recognized for transmitting dengue fever in many areas across the globe. Climate change has also contributed to mosquito range extension and pesticide resistance; hence, minimal insecticide exposure is suggested in anti-mosquito activities [41]. Personal protection by impregnating insecticides on a fabric is one of the successful methods for repelling and preventing bites from various hematophagous insects like mosquitoes, tsetse, mites, ticks, etc. [15, 18, 42–45]. The previous literature shows that pyrethroids can serve as a potent spatial repellant producing excito-repellency of mosquitoes and bed bugs. In fact, both ACP and DET at particular concentrations can elicit behavioral avoidance leading to inhibition of blood feed and eventually resulting in termination of the insects [42, 44, 46].

In this study, insecticides were impregnated on a polyester cotton (PC) blend fabric using the dipping method. The impregnated fabrics were evaluated for their potential in repelling bed bugs (*C. lectularius*) and mosquitoes (*Ae. aegypti* and *Ae. albopictus*) along with their physical properties at various time intervals after different cycles of washing. The results from in vivo assays were consistent with the previous findings. In the Petri plate assay, the bed bugs were given equal opportunity to move to two equal halves of the Petri plates containing untreated as well as impregnated fabric. The Petri plate assay using DEET-impregnated fabric performed by Wang et al. in 2013 showed this to be a fast and simple method for studying the repellency of bed bugs [47]. Many herbal and essential oil-based formulations are currently available on the market but due to their short duration of action (lasting up to 8–12 h) the applicability is limited; contrarily our developed IIF will be effective for a relatively longer period of time, up to a few months (minimum of 20 washing cycles) [48]. In the current study by utilizing a similar assay protocol, the repellency was found to be 80% after 24 h of exposure for unwashed fabric and gradually deceased to 56.6% after the 20th wash. A cone bioassay was performed adopting WHO guidelines (WHO 2005), which revealed the percentage knockdown and mortalities of mosquitoes up to 60 min and 24 h post exposure [38]. The percentage knockdown decreased after successive washes but was still capable of causing > 50% knockdown after the final wash. As compared to a previous study conducted by Sukumaran et al., in 2014, a 100% mortality rate was observed up to the 25th washing cycle, which further decreased after subsequent washes [49]. Contrarily, 83.3% mortality was seen in the present study against both species of mosquitoes, and the values were found to be non-significant (*P* > 0.05). The slight decrease in mortality rate can be attributed to the manual dipping method [18].

The residual analysis was performed to quantify the contents of insecticides remaining after different wash cycles. For this, the WHO procedure was adopted for washing, followed by ultrasonic extraction method. The extraction was performed at a constant temperature of 75 ºC with continuous stirring using acetonitrile as an extraction solvent. The temperature was chosen as such because of the presence of a binder (PVA) used in the fabric which melts above 70 ºC [50]. The optimized chomatographic conditions used in the study for individual insecticides are given in Additional file 1: Table S1. The chomatograms showed good separation peaks for both ACP and DET. From the results of insecticide residual analysis, 85.9% reduction in the content of DET and 40.7% reduction in the concentration of ACP were found after 20 washes. These reductions in the residual contents can correspond to the overall reduction of in vivo bioefficacy of IIF tested against both mosquitoes and bed bugs.

For the surface morphology, SEM and EDX analyses are considered to be the evaluation methods that can indicate successful impregnation [49, 51]. The SEM micrographs of control and IIF (Fig. 4) show clear deposition of insecticidal agents between the fibers. The elemental analysis of IIF performed using EDX (Fig. 5) further shows the identification lines for the large emission energies of elements as evident from the spectrum. As structural skeleton of ACP and DET contains chlorine, bromine, and nitrogen atoms in addition to carbon and oxygen. Thus, the appearance of these particular elements suggests the presence of alpha-cypermethrin (C_22_H_19_Cl_2_NO_3_) and deltamethrin (C_22_H_19_Br_2_NO_3_) being impregnated on the fabric. In the control sample, however, no such elements were observed.

The DSC-TGA analysis was performed to observe the changes in heat capacity resulting from endothermic and exothermic transitions along with the thermal degradation behavior of the control fabric and IIF. For IIF, a distinguishable endothermic peak was observed at 98.3 °C, which may be due to the presence of pyrethroids. From TGA thermograms, the initial mass loss was observed beyond 260 ºC, which may be due to initiation of polyester fiber melting. After this, two mass loss steps were observed, one at around 340–370 °C (40–50%), which may have been due to cotton fiber degradation, and the other at around 440–455 °C (70–80%), which was due to the final degradation of polyester fibers from the fabric samples [52]. As the temperature rose up to 600 °C, final weight loss was observed by 90–95%. Total weight losses of the samples were identified, and the decomposition of control fabric was found to be slightly lower than in the test. Thus, from the DSC-TGA analysis, the significant endothermic peak and weight loss pattern show successful impregnation and do not cause any hindrance to the thermal properties of the fabric [41].

Concerning the physical properties, the GSM values of the fabric increased after impregnation because the insecticides adhered to the surface of the fabric [53–56]. In the current study, the GSM value of unwashed IIF was found to be increased compared with the control sample. According to the ISO-specified requirement of standard pH, the direct use fabric should be within the range of 4.0–7.5 [35]. Higher or lower values not only affect the performance of the textile but also may harm human health. No such deviations of pH were observed in the fabric after impregnation, while the bursting strength, which is usually a determining parameter of specimen’s integrity, slightly decreased after each washing. Similarly, fabric stiffness was also observed to be decreased because of reduction of inter-yarn friction within the fabric. The binder used in the impregnation was known to cause a small reduction in the fiber lubrication, which was also observed in the present study [57]. The flexural rigidity of a fabric generally depends on the yarn properties. The initial increase in the value may be attributed to the deposition of insecticides and polymer solution, which reduces the internal friction of the fibers [58]. Additional decrease after subsequent washes can be due to the loss of active ingredients and fibers protruding from fabric surfaces [35].

## Conclusion

In conclusion, the insecticide-impregnated fabric (IIF) can serve as a potential barrier to pathogenic vectors with a good durability and long-lasting property. This may be attributed to the incorporation of pyrethroids in combination, which provides the optimum efficacy; this is conclusively supported by the values found from the present study. Future studies could be interesting to look for other target vectors to evaluate their repellant characteristics.

## Supplementary Information

**Additional file 1: Table S1.** HPLC chromatographic conditions for ACP and DET.

## Data Availability

All data generated or analyzed during this study are included in the final manuscript.

## Abbreviations

ACP: Alpha-cypermethrin
DET: Deltamethrin
IIF: Insecticide-impregnated fabric
HPLC: High-performance liquid chromatography
SEM: Scanning electron microscopy
EDX: Energy-dispersive x-ray
DSC: Differential scanning calorimetry
TGA: Thermo-gravimetric analysis
PC: Polyester cotton
PVA: Polyvinyl acetate
WHO: World Health Organization
RPM: Rotations per minute
GSM: Gram per square meter
ANOVA: Analysis of variance
DEET: N, N-Diethyl-meta-toluamide
ISO: International Organization for Standardization
