# Supplementary material for: Development of insecticide-impregnated polyester/cotton blend fabric and assessment of their repellent characteristics against Cimex lectularius and dengue vectors Aedes albopictus and Aedes aegypti
Source: Parasit Vectors. 2023 Apr 9;16:122. doi: 10.1186/s13071-023-05740-1 (PMC10082990; doi:10.1186/s13071-023-05740-1)
Supplement: Supplementary file 1 — Additional file 1: Table S1. HPLC chromatographic conditions for ACP and DET. [file 13071_2023_5740_MOESM1_ESM.docx]

**Additional file 1: Table S1** HPLC Chromatographic conditions for ACP and DET

| **Insecticides:** | **ACP** | **DET** |
| --- | --- | --- |
| **Mode** | Isocratic | Isocratic |
| **Column** | C_18_ reverse phase | C_18_ reverse phase |
| **Mobile phase** | Acetonitrile: water (90:10) | Acetonitrile: water (90:10) |
| **Flow rate** | 1mL/min | 1.2 mL/min |
| **Column temperature** | 27^o^C | 27^o^C |
| **Injection volume** | 10 µL | 10 µL |
| **Run time** | 7 min | 7 min |
| **Wavelength** | 277nm | 268nm |
